# ^99m^Tc标记T7肽及其在裸鼠非小细胞肺癌模型体内的生物分布研究

**DOI:** 10.3779/j.issn.1009-3419.2014.03.02

**Published:** 2014-03-20

**Authors:** 玉美 郝, 欣 贺, 晓靓 周, 爱民 孟, 鉴峰 刘, 金剑 刘, 娜玲 宋

**Affiliations:** 300192 天津，中国医学科学院北京协和医学院放射医学研究所，天津市放射医学与分子核医学重点实验室 Department of Tianjin Key Laboratory of Radiation Medicine and Molecular Nuclear Medicine, Institute of Radiation Medicine, Peking Union Medical College, Chinese Academy of Medical Sciences, Tianjin 300192, China

**Keywords:** ^99m^Tc-T7, 肿瘤抑素T7肽, 整合素αvβ3, 肺癌显像, 生物分布, ^99m^Tc-T7, Tumstatin T7 peptide, Integrin αvβ3, Lung cancer imaging, Biodistribution

## Abstract

**背景与目的:**

肺癌是一种死亡率极高的恶性肿瘤，针对肺癌的早期诊断和治疗有着重要的意义和价值，本研究旨在探讨羰基锝法标记的肿瘤抑素T7肽用作裸鼠肺癌早期显像剂的初步研究。

**方法:**

采用羰基锝法标记T7肽，薄层色谱法检测^99m^Tc-T7的放化纯度与稳定性，丙酮作展开系统。测定^99m^Tc-T7与NCI-H157细胞的亲和力。研究^99m^Tc-T7于0.5 h、1 h、2 h、4 h、8 h在荷人非小细胞肺腺癌裸鼠体内的生物分布特性，并计算肿瘤（T）与非肿瘤组织（NT）放射性比值。

**结果:**

^99m^Tc对T7肽标记率高，放化纯度达90%以上，不需进一步纯化，体外稳定性好。^99m^Tc-T7与NCI-H157细胞的平衡解离常数为196.1 nM。^99m^Tc-T7在裸鼠体内主要通过内脏器官代谢，血液清除较快，在肿瘤部位有一定程度聚集，肿瘤/肌肉比值随时间延长而增加，可达到5以上，在4 h-8 h之间取值较为理想。^99m^Tc-T7在肺内存在一过性聚集。

**结论:**

^99m^Tc-T7制备方法简便，标记率高，稳定性好，并能在肺癌肿瘤部位聚集，有望用作肺癌SPECT/CT显像剂。

## Abstract (en)

**Background and objective:**

Lung cancer is a malignant tumor with high mortality rates. This study aims to develop potential candidates of integrin αvβ3 imaging agents, which can facilitate the diagnosis and treatment of lung cancer.

**Methods:**

The T7 peptide was labeled with carbonyl technetium. The thin layer chromatography with acetone as the development system was performed to investigate the purity and stability of ^99m^Tc-T7. The binding affinity of ^99m^Tc-T7 with NCI-H157 tumor cells was determined. The biodistribution of ^99m^Tc-T7 in nude mice bearing non-small cell lung carcinoma was observed after injection of ^99m^Tc-T7 at 0.5 h, 1 h, 2 h, 4 h, and 8 h, and the radioactive ratio of tumor (T) and non-tumor tissues (NT) was calculated.

**Results:**

^99m^Tc labeled T7 had high radiochemical purity of more than 90%, which does not require further purification, with good stability in vitro. The association and dissociation constant (KD) of ^99m^Tc-T7 with NCI-H157 tumor cells was 196.1 nM. ^99m^Tc-T7 was mainly metabolism through the internal organs with rapid blood removal. Moreover, the uptake in tumor tissue was significantly higher than the muscle with tumor/muscle ratio of 5.8. In addition, the ^99m^Tc-T7 exhibited a transient accumulation in the lungs.

**Conclusion:**

The ^99m^Tc-T7 could be prepared using a simple method, had high labeling rate and good stability, and could be accumulated at tumor site. Thus, ^99m^Tc-T7 is a potential lung cancer SPECT/CT imaging agent.

肺癌是一种临床常见疾病，80%为非小细胞肺癌（non-small cell lung cancer, NSCLC），其发病率和死亡率已跃居恶性肿瘤首位，5年内生存率极低，肺癌的早期诊断对于降低肺癌患者死亡率和提高患者的存活时间极为关键^[1]^。目前在肺癌早期显像领域，^18^F-FDG PET/CT显像虽然取得了一定的临床效果，但由于其存在一定的假阳性率和假阴性率，PET/CT对于肿瘤的应用前景仍有赖于新型特异性更高的显像剂开发。目前研究发现，肿瘤新生血管内皮细胞及某些肿瘤细胞表面高表达整合素αvβ3，而已存在的血管和正常组织中并不表达或表达量很低，整合素αvβ3可作为肿瘤新生血管的重要标志物^[2]^，由于肿瘤组织新生血管丰富，整合素αvβ3成为了肿瘤诊断和治疗的有力靶点^[3]^，近年来关于αvβ3受体靶向诊断和治疗方面的研究已有许多报道^[4-6]^。肿瘤抑素（Tumstatin）是继内皮抑素（Endostatin）和血管生成抑素（Angiostatin）之后，源于人血管基膜Ⅳ型胶原蛋白α3链的肿瘤抑制因子，能够以RGD非依赖方式结合于整合素αvβ3，抗肿瘤活性区定位于T7片段区。本研究所采用的T7肽即是来源于肿瘤抑素Ⅳ型胶原蛋白的25肽，可通过RGD非依赖模式与整合素αvβ3特异结合并抑制肿瘤新生血管生成^[7]^。本研究旨在探讨将其用作肺癌靶向显像探针的可能性。T7分子量小，免疫原性低，结合位点明确，是一个非常有潜力的活性探针^[8]^。本研究采用羰基锝法标记T7肽，研究^99m^Tc-T7在荷人NSCLC（NCI-H157）裸鼠肿瘤模型体内的生物分布，探讨^99m^Tc-T7用作肺癌显像剂的可能性。

## 1 材料和方法

### 1.1 材料

NCI-H157人NSCLC细胞购自中国医学科学院肿瘤细胞库；BALB/c裸鼠25只（7 g-19 g），4周-6周龄，雄性，购自中国医学科学院实验动物研究所；T7肽（TMPFLFCNVNDVCNFASRNDYSKKK），分子量2, 942.41，纯度96.84%，委托北京亚美多肽生物科技有限公司合成；硼氢化钠、碳酸钠、酒石酸钾钠、HCl、丙酮由天津江天化工公司提供；高锝酸溶液（^99m^TcO_4_^-^）由北京原子高科股份有限公司提供；SN-6100型全自动放射免疫γ计数器（上海核所日环光电仪器有限公司）；薄层扫描仪AR-2000（BioScan）。

### 1.2 方法

#### 1.2.1 ^99m^Tc-T7的标记

羰基锝法标记多肽反应式见图 1。本研究通过计算机分子模拟手段分析T7与整合素αvβ3受体的结合特异性并将其结合活性位点定位于Ser90、Arg91、Asp93和Tyr94^[9]^，为改善T7肽水溶性，在不改变其活性和结合位点的前提下，将T7序列N端三个疏水氨基酸改为赖氨酸（Lys, K），分子量2, 942.41，纯度96.84%，序列为TMPFLFCNVNDVCNFASRNDYS KKK。精确称量硼氢化钠20 mg、碳酸钠4 mg、酒石酸钾钠15 mg，装入一个西林瓶中，密封后通入CO气体30 min，将瓶内空气排空压好铝盖后备用。将2.5 mL ^99m^TcO_4_^-^生理盐水淋洗液（74mBq，可根据实验需要调整活度与体积），用注射器注入西林瓶中，通入CO气体，80 ℃水浴反应15 min。反应结束后，西林瓶冷却至室温，加入1 M HCl调节pH至中性。将新鲜制备的^99m^Tc(CO)_3_^+^加入T7肽溶液中，50 ℃反应90 min。取出自然冷却至室温备用。

**1:**
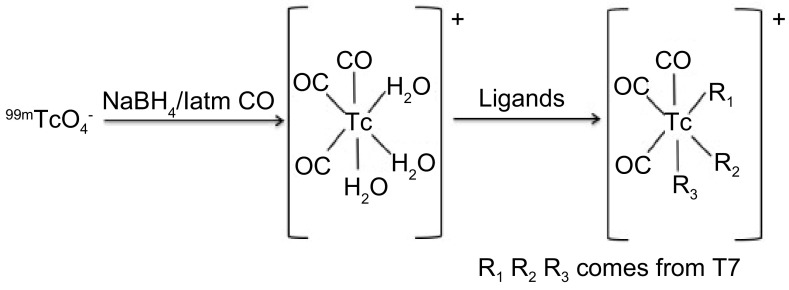
羰基锝法标记多肽反应式。R1、R2、R3代表来自于多肽游离氨基残基的-NH2或者-SH。 The equation of carbonyl technetium labeling polypeptide. R1, R2, R3 represens the -NH2 and -SH comes from the polypeptide amino free residues.

#### 1.2.2 ^99m^Tc-T7的放化纯与稳定性检测

薄层色谱法检测标记率及放化纯度，以硅胶板为固定相，丙酮作为展开系统，毛细管点样，放射性γ计数器进行检测。稳定性检测：取500 μL的^99m^Tc-T7溶液分别加入1 mL的生理盐水和1 mL人血清中，37 ℃孵育8 h，用薄层色谱法检测放化纯度。

#### 1.2.3 ^99m^Tc-T7与NCI-H157细胞亲和力测定

根据放射性配体受体结合测定方法来测定放射性配体^99m^Tc-T7与NCI-H157细胞表面整合素αvβ3受体的亲和力，本研究采用6浓度4孔平行实验。用含10%FBS的DMEM培养基培养NCI-H157细胞，并将其按照20, 000个/500 μL/孔铺2块24孔板，分别为总结合板和非特异性结合板（总结合代表^99m^Tc-T7与反应体系中所有可结合位点的结合；非特异性结合代表^99m^Tc-T7与整合素αvβ3之外的位点结合），培养过夜待用。将非特异性结合板的培养液弃去，500 μL冷PBS洗两次，加入200 μL未标记的80 mM T7液进行饱和，冰上孵育1 h。将^99m^Tc-T7配制成如下浓度：5 nM、10 nM、20 nM、40 nM、80 nM、160 nM；将总结合板和非特异性结合板内的培养液或冷物质均弃去，500 μL冷PBS洗2次，对应加入6个浓度的^99m^Tc-T7溶液，每个浓度设4个平行孔，冰上孵育1 h，将溶液弃去，500 μL冷PBS洗2次，加入1 mL 1 M NaOH消化，室温反应1 h，转移到计数管内，测量各孔的放射性计数，总结合计数与非特异性结合计数的差值即为特异性计数。数据处理，绘制放射性受体配体结合饱和曲线和Scatchard图，得到解离常数KD值。

#### 1.2.4 荷人NCI-H157裸鼠肺癌模型构建

将NCI-H157细胞株培养至对数生长期，弃去培养液，PBS清洗2次，0.25%胰酶消化，收集细胞，离心，生理盐水重悬并计数。无菌条件下，每只裸鼠右后肢背部皮下注射6×10^6^个/200 μL的NCI-H157细胞悬液。裸鼠置于天津市实验动物中心SPF级动物房饲养，当肿瘤长径达0.6 cm-1.0 cm时进行实验（约30 d）。

#### 1.2.5 ^99m^Tc-T7在荷人NCI-H157裸鼠体内的生物分布测定

将荷瘤裸鼠随机分成5组（0.5 h、1 h、2 h、4 h和8 h），每组5只，分别尾静脉注射3.7 MBq/100 μL的^99m^Tc-T7溶液，注射后分别在0.5 h、1 h、2 h、4 h和8 h股动脉放血处死，分别取心、肝、脾、肺、肾、肌肉、骨骼、脑、肿瘤、小肠、胃，称湿重，测量γ计数，计算各脏器的每克组织注射剂量百分比（%ID/g）并分析肿瘤/非肿瘤（T/NT）比值。

## 2 结果

### 2.1 ^99m^Tc-T7的制备及质控

^99m^Tc-T7制备后，用薄层色谱法测得高锝酸溶液的放化纯为98%，^99m^Tc-T7的放化纯为92.1%，见图 2。^99m^Tc-T7在37 ℃生理盐水和37 ℃人血清中放置8 h后，放化纯均仍高于90%，见图 3。结果表明^99m^Tc-T7标记率高且具有较好的稳定性。

**2:**
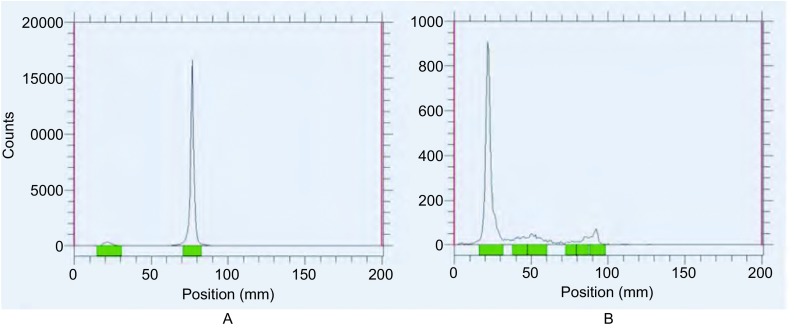
^99m^Tc-T7薄层色谱扫描图。A：高锝酸溶液，放化纯98%；B：^99m^Tc-T7，放化纯92.1%。 ^99m^Tc-T7 thin layer chromatography scan. A: high technetium acid solution, radiochemical purity 98%; B: ^99m^Tc-T7, radiochemical purity 92.1%.

**3:**
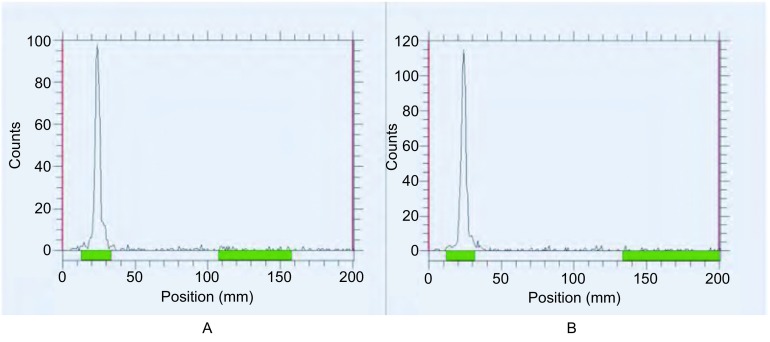
^99m^Tc-T7体外稳定性检测。A：^99m^Tc-T7在37 ℃生理盐水8 h后，放化纯 > 90%；B：^99m^Tc-T7在37 ℃人血清8 h后，放化纯 > 90%。 ^99m^Tc-T7 stability tests in vitro. A: ^99m^Tc-T7 with high radiochemical purity over 90% after 8 h at 37 ℃ saline water; B: ^99m^Tc-T7 with high radiochemical purity over 90% after 8 h at 37 ℃ human serum.

### 2.2 ^99m^Tc-T7与NCI-H157细胞的平衡解离常数分析

在放射性配基浓度^99m^Tc-T7足够大的情况下，受体整合素αvβ3全部被其结合，这时的放射性配基^99m^Tc-T7的结合量即反映出受点含量，我们将其称为最大结合量，以B_max_表示。若以B代表与受体整合素αvβ3结合的配基量，F代表配基^99m^Tc-T7的浓度，KD代表解离常数。根据Clark受体理论：B＝B_max_•F/(F+KD)，B与F作用是一条直角双曲线，将B＝B_max_•F/(F+KD)变形得：B/F＝B_max_/KD－B/KD，以B/F为纵坐标，B为横坐标，即为Scatchard作图，线性回归，斜率m=1/KD^[10]^。

^99m^Tc-T7与NCI-H157细胞的结合饱和曲线见图 4，Scatchard作图法分析结果见图 5。^99m^Tc-T7与NCI-H157细胞的KD值为131.6 nM。

**4:**
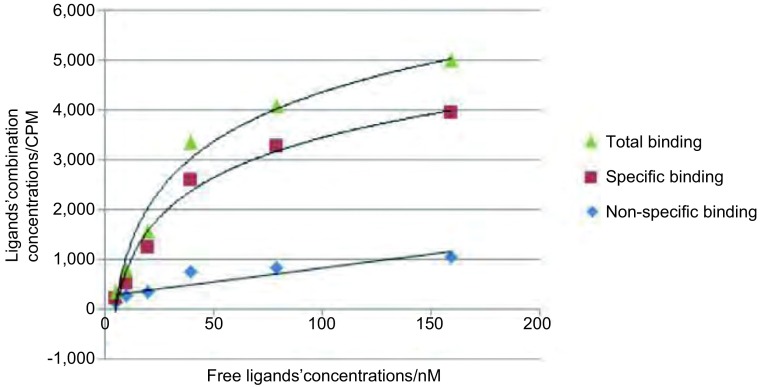
^99m^Tc-T7与NCI-H157细胞的亲和力测定，饱和曲线图 Affinity determination of ^99m^Tc-T7 and NCI-H157, Saturation curve graphs

**5:**
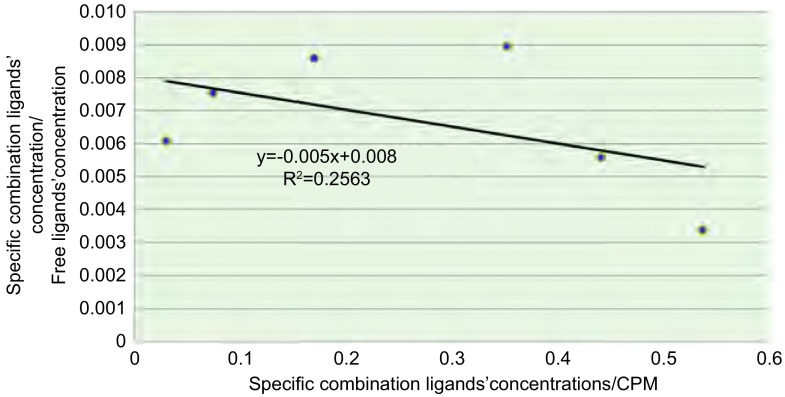
^99m^Tc-T7与NCI-H157细胞的亲和力测定，Scatchard作图 Affinity determination of ^99m^Tc-T7 and NCI-H157, Scatchard plot

### 2.3 ^99m^Tc-T7在裸鼠肺癌模型体内的生物分布

裸鼠肺癌模型注射^99m^Tc-T7后，从0.5 h-8 h内均显示出较高的肿瘤摄取，从注射后2 h-8 h肿瘤的放射性摄取均高于肌肉、骨骼、脑、小肠、胃等组织，见表 1和图 6。肿瘤/肌肉的T/NT比值随着时间延长而增加，能达到5以上，且在4 h-8 h之间取值较为理想，见图 7。此外，标记物在肺组织内存在一过性聚集，肺组织放射性强度在注射2 h后逐渐降低，见图 6，有必要进一步研究^99m^Tc-T7在肺部肿瘤的滞留性质，研究其在原位肺癌模型中的生物分布特征，探索其应用前景。脑放射性始终呈低水平，见图 6，表明^99m^Tc-T7不能正常通过血脑屏障，有可能用于评价血脑屏障的完整性，且要进行脑部肿瘤显像尚需对T7肽的结构进一步改造。据表 1中数据可见，^99m^Tc-T7在肺部和血液中的放射性水平明显高于肿瘤，该显像剂仍存在本底高这一普遍性问题，尚需对T7肽进行更深一步的改造，如引入双功能螯合剂（DTPA、DOTA、HYNIC及葡聚糖等），来加速组织本底放射性的降低，提高显像图像质量。注射^99m^Tc-T7后，肝、脾的放射性较高，肾脏次之，表明此标记物主要通过肝脾代谢，泌尿系统次之，且随时间延长血液的放射性摄取值（%ID/g）降低较快，8 h时血的放射性摄取值（%ID/g）可降为0.5 h时的26%，表明^99m^Tc-T7血液清除较快。

**1:** ^99m^Tc-T7在NCI-H157裸鼠模型体内生物分布（%ID/g±SD）（*n*=5） The biodistribution of ^99m^Tc-T7 in nude mice model (%ID/g±SD)(*n*=5)

Tissue	0.5 h	1 h	2 h	4 h	8 h
Heart	1.293, 1±0.092, 9	1.553, 6±0.103, 0	0.827, 9±0.038, 9	0.514, 7±0.011, 1	0.437, 7±0.019, 6
Live	1.830, 5±0.040, 9	2.375, 6±0.108, 5	2.237, 3±0.103, 8	2.201, 0±0.091, 5	2.176, 8±0.062, 6
Spleen	2.044, 2±0.046, 0	2.285, 9±0.123, 8	1.848, 2±0.058, 0	1.571, 9±0.060, 9	1.475, 4±0.074, 8
Lung	3.458, 0±0.113, 2	6.621, 3±0.168, 8	6.726, 0±0.110, 9	3.270, 0±0.104, 9	2.722, 8±0.055, 1
Kidney	0.491, 9±0.010, 2	1.536, 7±0.152, 8	0.861, 7±0.020, 4	0.667, 3±0.019, 1	0.537, 0±0.013, 8
Muscle	0.020, 9±0.001, 2	0.015, 3±0.000, 7	0.016, 9±0.000, 8	0.015, 9±0.000, 8	0.016, 5±0.000, 7
Bone	0.026, 0±0.002, 2	0.054, 7±0.002, 2	0.034, 9±0.001, 3	0.031, 6±0.001, 0	0.025, 6±0.000, 3
Brain	0.008, 4±0.000, 5	0.008, 1±0.000, 2	0.007, 3±0.000, 3	0.008, 1±0.000, 6	0.007, 9±0.000, 1
Tumor	0.025, 6±0.000, 6	0.035, 9±0.001, 0	0.086, 5±0.002, 3	0.078, 5±0.004, 1	0.073, 7±0.003, 2
Blood	0.928, 4±0.010, 0	0.770, 6±0.020, 5	0.669, 2±0.026, 7	0.553, 0±0.018, 4	0.245, 9±0.009, 8
Intestine	0.049, 8±0.001, 1	0.052, 8±0.003, 4	0.045, 2±0.002, 1	0.047, 1±0.001, 2	0.035, 1±0.001, 8
Stomach	0.086, 2±0.004, 2	0.088, 0±0.003, 6	0.077, 5±0.005, 8	0.077, 3±0.002, 4	0.071, 9±0.004, 3

**6:**
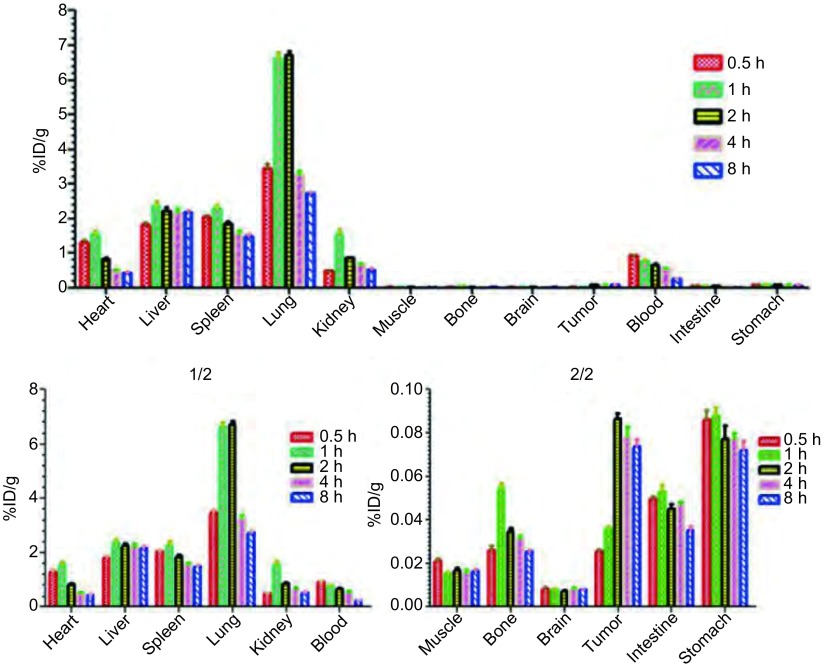
^99m^Tc-T7在荷人NCI-H157裸鼠异位模型体内生物分布（%ID/g） The biodistribution of ^99m^Tc-T7 in NCI-H157-bearing nude mice model (%ID/g)

**7:**
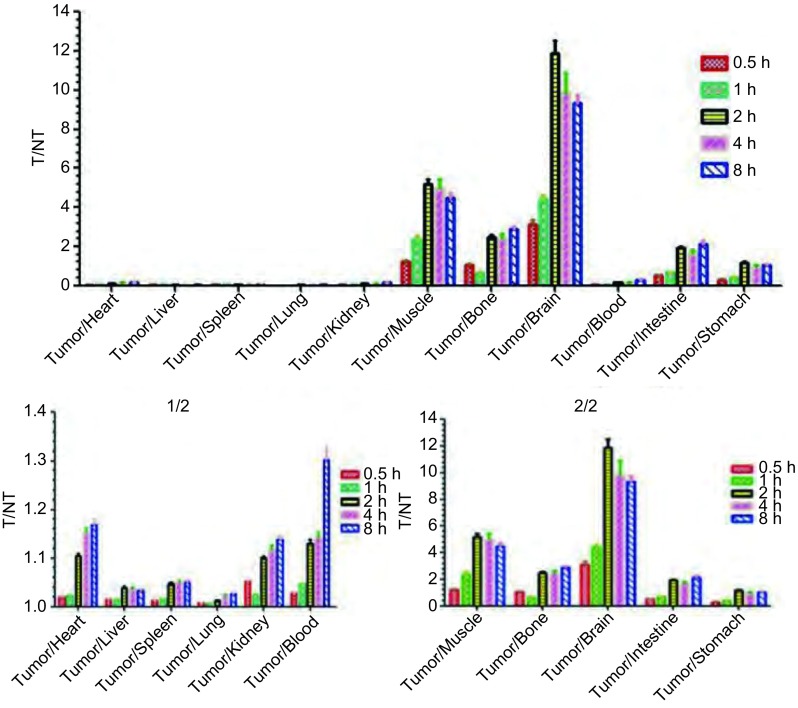
^99m^Tc-T7在荷人NCI-H157裸鼠异位模型分布特异性分析（T/NT） Specificity analyzing of the ^99m^Tc-T7 biodistribution in NCI-H157-bearing nude mice model (T/NT)

## 3 讨论

肺癌已位居世界癌症死亡率的首位，其早期诊断对降低肺癌的死亡率和提高患者存活时间至关重要，早期肺癌手术切除后的5年生存率可达到67%-80%以上。目前临床常用的检查手段主要有：胸部CT、纤维支气管镜检查、血清学检查等^[11]^，但都存在一定的局限性，大多数患者在诊断出肺癌时已发生了转移，且有40%的NSCLC已发生远处转移^[12]^，随着现代医学的发展，如何快速、早期、精确、无创地诊断肺癌，对影像医学提出了更高的要求。目前在肺癌PET/CT无创显像领域已应用到临床的显像剂是^18^F-FDG，它是一种葡萄糖的类似物，恶性肿瘤因局部缺氧和肿瘤生物学行为使其代谢速率异常增高，耗糖量增加，^18^F-FDG即可通过细胞膜上的葡萄糖载体进入细胞内而显像。但由于^18^F-FDG并非肿瘤的特异性显像剂，肺炎、支气管扩张、活动性肺结核和肉芽肿等的炎症反应也可以摄取^18^F-FDG，造成^18^F-FDG PET/CT高达10%左右的假阳性率，导致一部分患者失去早期手术机会^[13]^。此外，由于部分分化较好，生长缓慢的肿瘤组织因对葡萄糖的依赖性低、糖代谢异常不明显，还会导致PET/CT显像一定程度的假阴性率^[14]^。基于上述原因，尚需发现更新更特异的肺癌诊断显像剂来提高肺癌诊断的准确率。

已有研究^[15]^表明，αvβ3整合素在肿瘤新生血管增生显像方面具有极大潜力，此外，αvβ3整合素靶向药物的研究也已处于临床Ⅰ期和Ⅱ期，据此，Beer等^[16]^将其研制的αvβ3靶向药物^18^F-galacto-RGD与基于葡糖糖代谢的^18^F-FDG进行了对比，虽未表明两者间存在一定关系，但在一良性支气管肿瘤患者的原位及转移部位发现^18^F-galacto-RGD的摄取值明显高于^18^F-FDG，结果表明αvβ3受体显像剂对于^18^F-FDG摄取值较低的良好肿瘤的显像诊断具有极大优势。本研究所采用的T7肽，亦是源于靶向αvβ3受体的肿瘤抑素Tumstatin上的一个有效作用片段，T7肽分子量小，免疫原性高，靶向性强，基于^99m^Tc-T7可以与肿瘤新生血管和部分肿瘤细胞表面高表达的αvβ3整合素结合来诊断肺癌，^99m^Tc-T7对炎症的亲和力低，且能发现良性肿瘤病灶，可以与^18^F-FDG联合显像提高肺癌诊断准确率，避免患者错失早期手术机会，^99m^Tc-T7有望成为肺癌SPECT/CT显像的早期特异性显像剂，降低肺癌早期诊断中存在的假阳性率和假阴性率，提高患者的存活率和存活时间。

目前多肽和蛋白质的放射性标记方法有很多，^125^I、^131^I、^3^H、^18^F、^99m^Tc、^72^As、^188^Re、^75^Br、^86^Yt等，由于^99m^Tc具有适宜的半衰期（*T_1/2_*=6.01 h）和γ光子能量（140 keV），它是SPECT显像的最佳核素；此外，^99m^Tc以^99m^TcO_4_^-^的化学形态很容易从^99^Mo-^99m^Tc发生器用生理盐水淋洗得到，价格低廉；它还可以和各种配体形成种类繁多、具有多种化学价态和不同生物分布性质的放射性药物，目前已合成的^99m^Tc-药物几乎能对人体所有脏器进行显像，以活体内各类生物大分子为靶目标，进行分子水平的显像，观察它在体内的吸收、分布、代谢过程，加速新药的开发^[17]^。羰基锝标记方法操作简便，标记率高，产物稳定性好。

本研究所得的^99m^Tc-T7放化纯高于90%，在37 ℃生理盐水和37 ℃人血清中放置8 h后，放化纯均仍高于90%，稳定性高。通过体外细胞亲和力实验发现，在暂无肿瘤新生血管增生的情况下，^99m^Tc-T7与NCI-H157细胞的KD值已达到131.6 nM的水平。通过^99m^Tc-T7在裸鼠肺癌模型体内生物分布研究发现^99m^Tc-T7对αvβ3受体具有高度的选择性和亲和力，在荷瘤鼠体内的肿瘤/肌肉放射性比值（T/NT）可达到5以上，在4 h-8 h之间取值较为理想，研究还发现^99m^Tc-T7在肺内存在一过性聚集。此外，^99m^Tc-T7主要通过内脏器官代谢，血液清除较快，对其进行适当修饰后有望用作肺癌SPECT/CT显像剂，用于肺癌的早期鉴别诊断、肿瘤分期、指导肺癌治疗方案的修订与修改、勾画放射性治疗靶区、判断预后疗效、检测肺癌的术后残余和复发，还利于寻找远处肺癌转移灶，穿刺活检定位等。本文对^99m^Tc-T7在肺癌显像领域的应用做了初步探讨，其是否能用做肺癌SPECT/CT显像剂还有待于其在SPECT/CT显像、药代动力学等方面展开更深入的研究。

## References

[1] Ren GH, Fan YG, Zhao YC (2013). Advance of lung cancer screening with low-dose spiral CT. Zhong guo Fei Ai Za Zhi.

[2] Liu Y, Yang Y, Zhang C (2013). A concise review of magnetic resonance molecular imaging of tumor angiogenesis by targeting integrin αvβ3 with magnetic probes. Int J Nanomed.

[3] Wu H, Chen H, Sun Y (2013). Imaging integrin α(v)β(3) positive glioma with a novel RGD dimer probe and the impact of antiangiogenic agent (Endostar) on its tumor uptake. Cancer Lett.

[4] Zhou Y, Shao G, Liu S (2012). Monitoring Breast tumor lung metastasis by U-SPECT-Ⅱ/CT with an integrin α(v)β(3)-targeted radiotracer(^99m^)Tc-3P-RGD(2). Theranostics.

[5] Liu S, Hsieh WY, Jiang Y (2007). Evaluation of a (^99m^)Tc-labeled cyclic RGD tetramer for noninvasive imaging integrin alpha(v)beta3-positive breast cancer. Bioconjug Chem.

[6] Zhang FH, Meng ZW, Tan J (2012). Radionuclide molecular target therapy for lung cancer. Int J Radiat Med Nucl Med.

[7] Naling S, Xin H, Qiren Z (2009). Cloning and expression of the tumstatin active peptides-T(7) and its derivant-T(7)-NGR. Clin Exp Med.

[8] He X, Zhao QR, Song NL (2007). Advance in research on tumstatin active peptides and their derivatives. Yao Xue Fu Wu Yu Yan Jiu.

[9] Zan JH, He X, Long W (2012). Insights into binding modes of tumstatin peptide T7 with the active site of αvβ3 integrin. Mol Simulat.

[10] Zhu QL (1986). Scatchard plot methods and its parameters solution. Zhongguo Yi Xue Ke Xue Yuan Xue Bao.

[11] Wang WJ, Tao Z, Gu W (2013). Clinical observations on the association between diagnosis of lung cancer and serum tumor markers in combination. Asian Pac J Cancer Prev.

[12] Reck M, Heigener DF, Mok T (2013). Management of non-small-cell lung cancer: recent developments. Lancet.

[13] Fujimoto R, Higashi T, Nakamoto Y (2006). Diagnostic accuracy of bone metastases detection in cancer patients: comparison between bone scintigraphy and whole-body FDG-PET. Ann Nucl Med.

[b14] 14Imai K, Minamiya Y, Saito H, et al. Diagnostic imaging in the preoperative management of lung cancer. Surg Today, 2013. [Epub ahead of print]

[15] Wu H, Chen H, Sun Y (2013). Imaging integrin alpha(v)beta(3) positive glioma with a novel RGD dimer probe and the impact of antiangiogenic agent (Endostar) on its tumor uptake. Cancer Lett.

[16] Beer A J, Lorenzen S, Metz S (2008). Comparison of integrin alphaVbeta3 expression and glucose metabolism in primary and metastatic lesions in cancer patients: a PET study using ^18^F-galacto-RGD and ^18^F-FDG. J Nucl Med.

[b17] 17Liu BL, Jia HM. Technetium radiopharmaceutical chemistry and its applications. 1^st^ end. Beijing: Normal university Publication, 2006. 56-57.刘伯里, 贾红梅主编. 锝药物化学及其应用. 第1版. 北京: 北京师范大学出版社, 2006. 56-57

